# Oxygen and Mortality in COVID-19 Pneumonia: A Comparative Analysis of Supplemental Oxygen Policies and Health Outcomes Across 26 Countries

**DOI:** 10.3389/fpubh.2021.580585

**Published:** 2021-07-13

**Authors:** Fatma Mansab, Harry Donnelly, Albrecht Kussner, James Neil, Sohail Bhatti, Daniel K. Goyal

**Affiliations:** ^1^COVID-19 Public Health Team, Public Health Gibraltar, Gibraltar, Gibraltar; ^2^Postgraduate School of Medicine, University of Gibraltar, Gibraltar, Gibraltar; ^3^Acute General Medicine, St Bernard's Hospital, Gibraltar Health Authority, Gibraltar, Gibraltar; ^4^Emergency Medicine, St Bernard's Hospital, Gibraltar Health Authority, Gibraltar, Gibraltar; ^5^Centre for Nutrition Education and Lifestyle Management (CNELM), London, United Kingdom; ^6^Clinical Lecturer, Postgraduate School of Medicine, University of Gibraltar, Gibraltar, Gibraltar

**Keywords:** COVID-19, SARS-CoV2, oxygen, mortality, treatment, early intervention, rationing, target oxygen saturation

## Abstract

**Introduction:** Hypoxia is the main cause of morbidity and mortality in COVID-19. During the COVID-19 pandemic, some countries have reduced access to supplemental oxygen, whereas other nations have maintained and even improved access to supplemental oxygen. We examined whether variation in the nationally determined oxygen guidelines had any association with national mortality rates in COVID-19.

**Methods:** Three independent investigators searched for, identified, and extracted the nationally recommended target oxygen levels for the commencement of oxygen in COVID-19 pneumonia from the 29 worst affected countries. Mortality estimates were calculated from three independent sources. We then applied both parametric (Pearson's R) and non-parametric (Kendall's Tau B) tests of bivariate association to determine the relationship between case fatality rate (CFR) and target SpO_2_, and also between potential confounders and CFR.

**Results:** Of the 26 nations included, 15 had employed conservative oxygen strategies to manage COVID-19 pneumonia. Of them, Belgium, France, USA, Canada, China, Germany, Mexico, Spain, Sweden, and the UK guidelines advised commencing oxygen when oxygen saturations (SpO_2_) fell to 91% or less. A statistically significant correlation was found between SpO_2_ and CFR both parametrically (*R* = −0.53, *P* < 0.01) and non-parametrically (−0.474, *P* < 0.01).

**Conclusion:** Our study highlights the disparity in oxygen provision for COVID-19 patients between the nations analysed. In those nations that pursued a conservative oxygen strategy, there was an association with higher national mortality rates. We discuss the potential reasons for such an association.

## Introduction

SARS-CoV2 causes COVID-19 (Coronavirus Disease 2019). As of May 2020, the total reported cases of COVID-19 were over 5 million, with 350,000 deaths over 5 months (1). More than half these deaths have occurred in the last month. Whilst there has been a slight reduction in the rate of growth for new infections globally, this is most likely due to strict infection control policies (e.g., case isolation, social distancing, and “lockdown”) (2). With the seroprevalence of SARS-CoV2 being reported as between <1 and 22% (3), it is most likely the majority of infections are yet to come, and the rate of infections will once again increase as infection control measures are balanced with economic pressures.

The true COVID-19 mortality rate is difficult to ascertain during the outbreak. Background infections, asymptomatic infections, testing criteria, reporting of fatalities, and the time lag between new cases and outcome are all potential confounders (4). This makes measuring the effects of national interventions difficult. It is reasonable, however, to expect that a nation's COVID-19 mortality rate will depend on access to healthcare and likely will also depend on the type of healthcare offered. The need for effective healthcare can be reasonably inferred from the marked disparity between mortality rates during a surge of cases vs. mortality post-surge (5).

Oxygen is a cornerstone of treatment for patients with COVID-19 pneumonia. Indeed, the major mechanism for injury and death in COVID-19 relates to hypoxia (6). It has been established that a delay in identifying and correcting hypoxia in pneumonia leads to increased disease severity, increased rate of mechanical ventilation, and increased mortality (7, 8). Whilst there are no controlled studies in COVID-19 specifically examining duration spent hypoxic and subsequent disease burden and mortality, Sun et al. have reported a reduction in the need for mechanical ventilation where hypoxia was detected and corrected early in patients with COVID-19 (9).

In relation to conservative oxygen strategies generally, there are four key mortality studies. The IOTA meta-analysis published in 2018 examined conservative vs. liberal oxygen strategies across a range of studies. None of the studies analysed related to pneumonia, and the majority of studies examined oxygen as a treatment not as a means to correct hypoxia. The authors suggest that optimal target oxygen saturations (SpO_2_) for all acute medical patients “might” be 94–96% (10).

Since the IOTA study, there have been three clinical studies, two of which were randomised controlled trials (RCTs), examining the mortality effect of conservative oxygen strategies. The ICU-ROX trial suggests that there may be no mortality effect at the higher target levels of SpO_2_ (actual mean SpO_2_ 96–97% vs. 95–96%) in mechanically ventilated patients from any cause (*n* = 1,000) (11). Another, a retrospective analysis, published in March 2020 examined over 35,000 intensive care patients and found the optimum SpO_2_ target of 94–98%. The authors note that patients who were in the optimal range for only 40% of the time had nearly twice the mortality of those who spent 80% of the time within the optimal target, even after correction for disease severity (12).

The most recent RCT, and the most well-controlled study of true conservative oxygen strategies to date (and the most relevant to COVID-19), examined 204 patients with acute respiratory distress syndrome (ARDS). Patients were randomised to either a conservative arm (actual SpO_2_ of 92–93%) vs. a liberal arm (SpO_2_ of 95–97%) and then followed up for 90 days. The study was halted early due to excessive deaths in the conservative oxygen group. In those patients with ARDS who were managed with a conservative oxygen strategy, there was a 27% increase in intensive care deaths and a 50% increase in 90-day mortality (13).

Despite the critical nature of oxygen therapy in COVID-19 pneumonia, there remains a marked variation between national guidelines for when to offer supplemental oxygen. Many nations seem to have implemented conservative oxygen strategies during the pandemic, effectively limiting the access of patients to supplemental oxygen. Others seem to have actively increased their capacity to offer supplemental oxygen for patients with COVID-19 pneumonia. Here, we examine the national guidelines from 29 nations to ascertain whether the national decision to promote a “conservative” oxygen strategy has any relationship to that nation's case fatality rate (CFR).

## Methods

We followed the advice for global reporting on health estimates as per the GATHER statement (14). All countries with more than 20,000 cases as of May 18, 2020, were assessed. Three investigators (DG, AK, and HD) independently identified the specific national recommendations for the target SpO_2_ to commence oxygen in patients with COVID-19. Two investigators (AK and HD) were blinded as to the reason for the study. Each nation's ministry of health, national guideline bodies, respiratory medicine bodies, and national health service were searched for relevant COVID-19 clinical guidelines. The European Society of Respiratory Medicine (15) was a useful resource with direct links to a number of COVID-19-specific clinical guidelines from across the world. Literature databases were also used as a means of identifying links to national guidelines. If guidelines were not available in one of the languages spoken by the investigators, online translation services were utilised, specifically for guidelines on “supplemental oxygen” or “oxygen therapy”—the entire guideline was not translated. Note, only guidelines applicable to the majority of the population were extracted, and guidelines for patients with underlying conditions such as chronic obstructive airways disease were not recorded.

**Figure 1:**
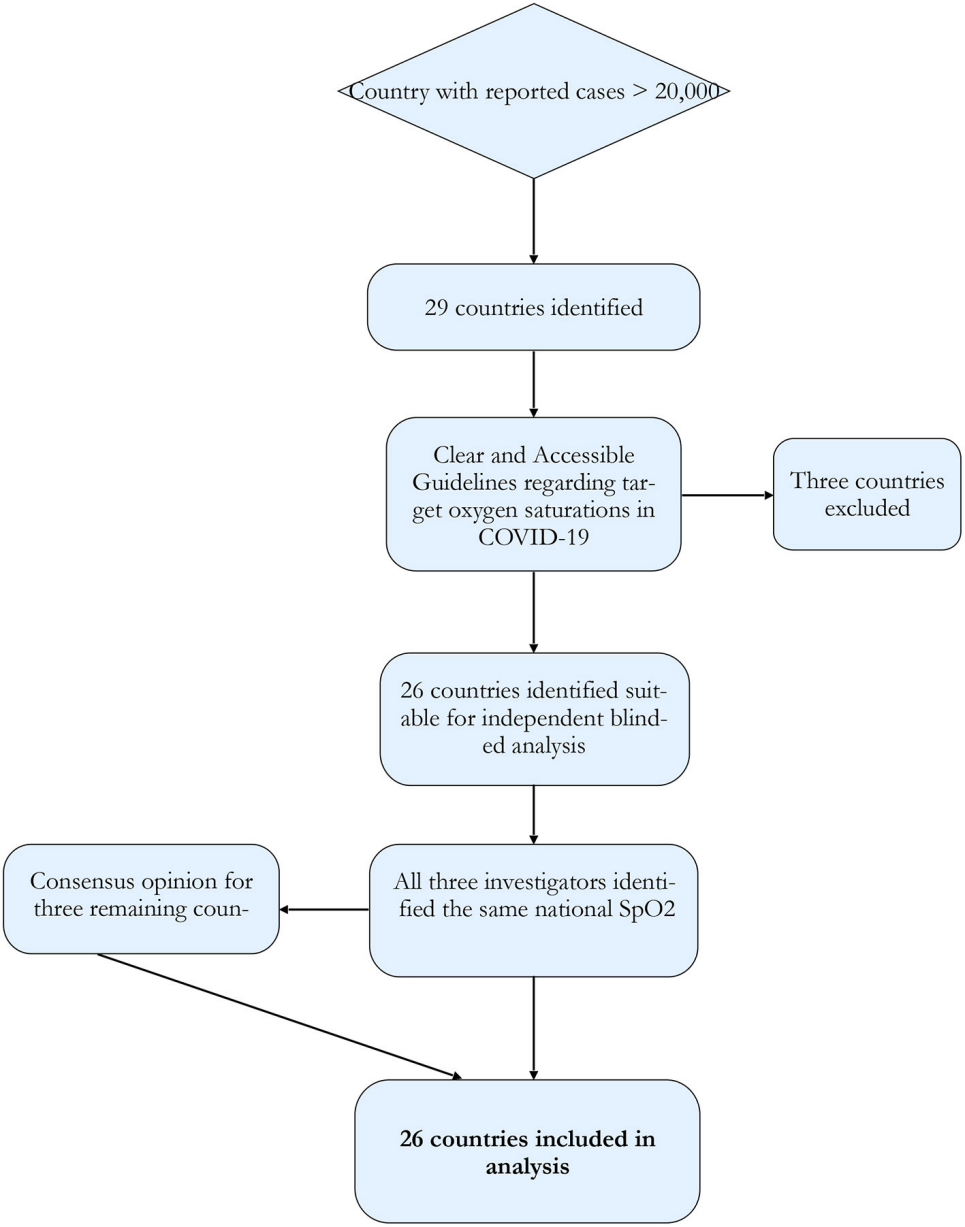
Study protocol flowchart. Using the WHO situation report countries with case numbers over 20,000 were selected. These countries were subjected to analysis *via* three different, independent investigators for the ascertainment of nationally recommended target oxygen saturations.

If guidelines were unclear, instruction was to disregard the country from further analysis. Where there was more than one recommendation, the investigator made a determination as to the most likely guideline to be followed (Figure 1). Where there was divergence between the three investigators, the consensus value was used. Results were tabulated and compared.

### CFR and Infection Fatality Rate

CFR is the percentage ratio of deaths to total cases. It is a crude figure privy to a number of potential confounders. For most nations, it is likely to be numerically incorrect (4). CFR, however, is likely to maintain a relationship to actual infection mortality rate (IFR) (3, 4) and as such was used in this study. CFR was calculated and cross-referenced from three different sources—The WHO, John Hopkins University, and Worldometer. There was no significant difference between the calculated CFR across the three sources.

### Statistical Analysis

All statistics were performed using SPSS. Both parametric (Pearson's R) and non-parametric (Kendall's Tau B) tests of bivariate association were performed to identify and characterised a potential trend between CFR and target SpO_2_, as given the small sample size it was not clear whether the assumptions of linearity, homoscedasticity, and normality of variables were met. Kendall's Tau B was used as the test of non-parametric bivariate association as there were ties in both CFR and SpO_2_, which are problematic for Spearman's Rho.

Scatter plots with linear regression lines of best fit are also given, as well as the y-intercept and gradient of these lines reported. Means and standard errors of CFR, SpO_2_, and confounding variables are given.

## Results

In total, there were 29 countries with a total case number over 20,000 on May 18, 2020. Of those, 26 countries had accessible clinical guidelines referring to target oxygen levels for the commencement of supplemental oxygen in COVID-19. UAE (United Arab Emirates) was excluded from further analysis as the national guidelines advised (at page 9) admitting all patients with COVID-19 to hospital, and commencing oxygen when “needed” (16). The Netherlands and Belarus were also excluded due to all three investigators failing to find clear national guidelines regarding oxygen targets.

Of the remaining 26 countries, there was concordance between all three investigators identifying the same national target oxygen levels in 23 countries. Of the remaining three countries (UK, Pakistan, and Qatar), determination of national target SpO_2_ in COVID-19 was made by consensus. For links to national guidelines, see Supplementary Material.

**Figure 2:**
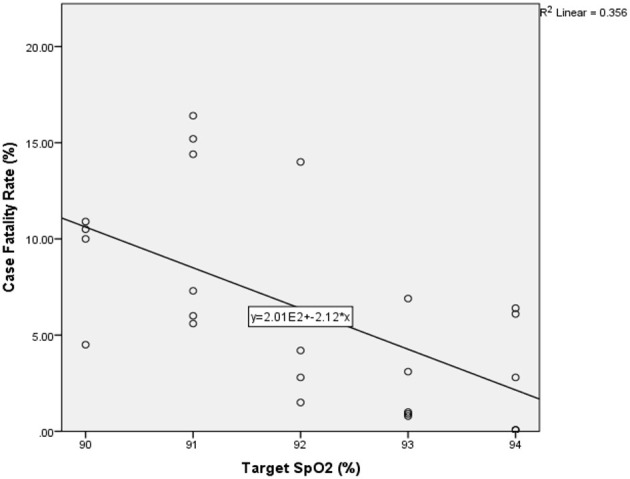
Scatter graph of national target oxygen saturations vs. national case fatality rate with best fit linear line (*n* = 26). SpO_2_–oxygen saturations.

**Figure 3:**
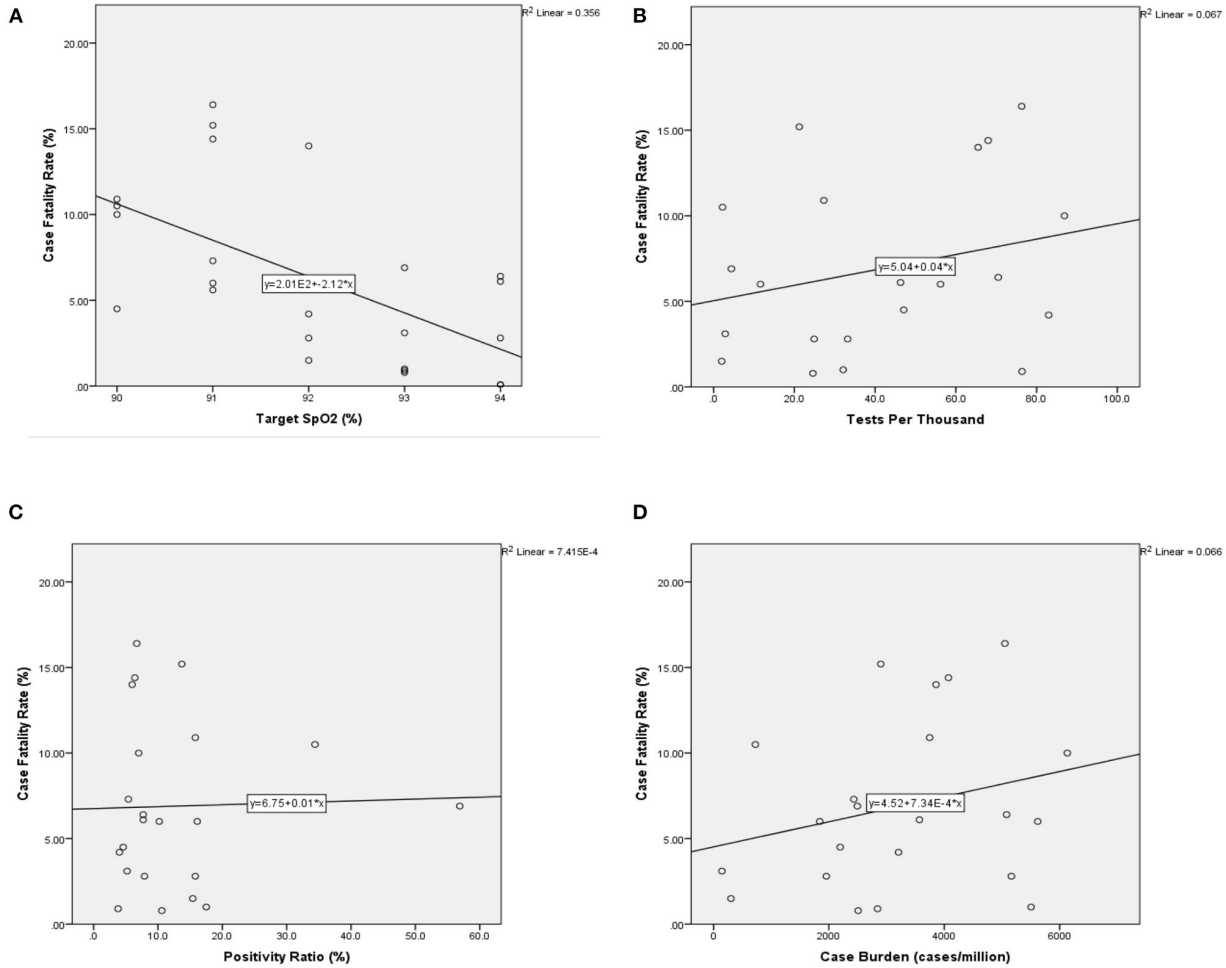
Scatter graphs with best-fit line for primary measure and confounders vs. CFR (*N* = 26). CFR is along the vertical axis in each and measured as percentage ratio. **(A)** Comparison of national CFR vs. nationally recommended target SpO2 (%) in COVID-19. **(B)** CFR vs. burden of cases measured by total number of cases per million inhabitants. **(C)** CFR vs. Testing Rate measured by total number of tests undertaken per thousand inhabitants (tests/thousand). **(D)** CFR vs. the overall positivity ratio (%), measured as the percentage ratio of positive tests to total tests. CFR, case fatality rate; SpO_2_, oxygen saturations. CFR, case fatality rate; SpO_2_, oxygen saturations.

Of the 26 nations analysed, six recommended commencing oxygen if SpO_2_ fell to below 95% (Singapore, Peru, Switzerland, Ireland, Qatar, and Pakistan), five made recommendation for below 94% (Saudi Arabia, Chile, Brazil, India, and Russia), five for below 93% (Portugal, Iran, Turkey, Bangladesh and Italy), six for below 92% (Canada, Belgium, France, UK, USA, and China), and four for below 91% (Germany, Mexico, Spain, and Sweden). CFR ranged from 0.06% (Qatar) to 16.4% (Belgium). A statistically significant correlation was found between SpO_2_ and CFR both parametrically (*R* = −0.53, *P* < 0.01) and non-parametrically (−0.474, *P* < 0.01), and no statistically significant correlation was found with the potential confounders analysed here. A scatter graph with the linear best-fit line is shown in Figures 2, 3A.

### Confounders

National guidelines for target saturations were relatively clear for most countries. Together with the high rate of consensus amongst investigators, it seems unlikely that investigator bias was a significant factor. The main confounders are more likely to stem from the many variables associated with CFR.

We found no correlation between CFR and cases/million inhabitants (Figure 3B), or tests/thousand inhabitants (Figure 3C), or overall positivity rate (Figure 3D), suggesting that the testing strategy between the countries examined did not have a significant visible relationship with our mortality measure, CFR (Table 1). We could not examine the potential impact of national-level reporting bias on the CFR from the data available.

**Table 1:** National CFR, target SpO_2_, and potential testing confounders in 26 countries.

	**SpO_**2**_**	**Tests per thousand**	**Positivity ratio**	**Case burden**	**CFR**
	**(%)**		**(%)**	**(cases/million)**	**(%)**
Qatar	94	82.3	25.9	20,311	0.06
Singapore	94	57.2	10.5	6,036	0.08
Pakistan	94	2.6	12.9	329	2.1
Peru	94	33.2	15.8	5,163	2.8
Switzerland	94	46.3	7.7	3,569	6.1
Ireland	94	70.6	7.7	5,080	6.4
Saudi Arabia	93	24.6	10.6	2,506	0.79
Russia	93	76.4	3.8	2,843	0.9
Chile	93	32.1	17.5	5,505	1
India	93	2.9	5.2	144	3.1
Brazil	93	4.4	56.9	2,492	6.9
Bangladesh	92	2.0	15.4	301	1.5
Turkey	92	24.9	7.9	1,955	2.8
Portugal	92	83.0	4	3,206	4.2
Iran	92	11.6	16.1	1,841	6
Italy	92	65.5	6	3,857	14
China	91	N/A	N/A	N/A	5.6
USA	91	56.2	10.2	5,620	6
Canada	91	45.7	5.4	2,431	7.3
UK	91	68.0	6.4	4,072	14.4
France	91	21.2	13.7	2,899	15.2
Belgium	91	76.3	6.7	5,051	16.4
Germany	90	47.1	4.6	2,194	4.5
Spain	90	86.9	7	6,133	10
Mexico	90	2.2	34.4	725	10.5
Sweden	90	27.3	15.8	3,746	10.9
Mean (Standard Error)	91.9 (1.32)	41.3 (28.3)	12.7 (12.1)	3,242 (1,722)	6.8 (4.8)
Correlation with CFR (Pearson's R)	**−0.53**	0.121	−0.026	−0.174	
2-tailed (*p*-value)	** <0.01**	0.57	0.91	0.42	

*CFR, case fatality rate; SpO_2_, oxygen saturations. Overall positivity ratio—percentage ratio of positive to total cases. Case burden—total number of positive cases per million inhabitants. Data extracted from WHO, Worldometer and John Hopkins University. Numbers in bold indicate a statistically significant result*.

## Discussion

National guidelines for when to commence supplemental oxygen in patients with COVID-19 varied significantly between the countries examined. Combined, the target SpO_2_ for the commencement of oxygen and target SpO_2_ for ongoing treatment varied from 90% to 98%. There are a number of potential reasons for the variation in oxygen policies between countries and the association with mortality.

### Causative Effect

Based on the design of the study, a causal relationship between national oxygen targets and national CFR cannot be determined. However, our analysis did reveal an association between national target oxygen saturations and national CFR—the lower the national target oxygen saturations, the higher the national CFR. One possible explanation for the identified relationship is that the national guidelines have been followed and implemented, and it is the lower oxygen levels during COVID-19 pneumonia that has led to an increased mortality.

Based on our current understanding of the effects of hypoxia on inflammation (17) and coagulation (18), there is good scientific basis for the increased mortality associated with suboptimal oxygen strategies and/or a delay in correcting hypoxia. There are direct effects of hypoxia leading to increased mortality, such as cardiac arrhythmias and ischemia-related pathologies [as identified in the aforementioned ARDS study (13)]. It is also quite plausible, indeed quite likely, given that hypoxia is pro-inflammatory, the delay in correcting hypoxia leads to more severe disease. This of course raises the possibility that rationing, or a conservative oxygen approach, or a failure to provide access to supplemental oxygen in COVID-19 pneumonia, actually increases healthcare burden and resource consumption (19).

The presence of “silent hypoxia”—low oxygen levels without respiratory distress—is likely to compound the mortality effect of reduced access to supplemental oxygen. A fall below normal SpO_2_ (95% or less) indicates progression of COVID-19 to pneumonia or pneumonitis. If sent home at this stage despite evidence for disease progression, around one-third of patients will be unaware of their own deterioration and therefore will either fail to re-present and demise at home, or will present even later (20). This will almost certainly add greater pressure on ICU facilities and increase morbidity and mortality. Conservative oxygen strategies are questionable at the best of times (19); with COVID-19, such strategies likely carry even greater harm. Optimal oxygen strategies have the additional benefit of identifying and observing these at-risk “silent hypoxia” patients.

### Resource Limitations

An alternative explanation for the relationship between national oxygen targets and mortality is that the recommendation to conserve oxygen simply reflects the resource limitations of the nation, and it is this resource limitation that causes an increase in mortality. For example, the UK directive to ration oxygen supply in April 2020 reduced the normal national target for the commencement of oxygen from SpO_2_ of 94% to a new value of 91%. The reason for rationing was related to the surge of infections and subsequent concern over the supply of oxygen (21). If such practises are common in other nations, the relationship between national guidelines' SpO_2_ and national CFR identified here may be a representation of the demands on healthcare during a surge of COVID-19 cases.

There are a number of reasons why mortality increases during a surge of infections. Patients are less likely to attend hospital or seek medical care, for fear of either contracting COVID-19 or overburdening their health service (22). Triage systems during a surge can be set with high thresholds for onward referrals (23). Another mortality factor is a potential lack of resources both staff and consumables. The overall delay to treatment that ensues prevents early correction of hypoxia, implementation of VTE (venous thromboembolism) prophylaxis, readjustment of medications (e.g., nephrotoxics) and the detection of secondary bacterial infection, and thus a likely increased mortality (24).

So then, the association between target SpO_2_ and CFR identified here may be more related to target SpO_2_ being an indicator of an overwhelmed healthcare service.

### National Approach

The issuing of national guidelines recommending lower target oxygen saturations than would be typical for viral pneumonias (25) may relate more to the overall approach of a national response to COVID-19, and as such, it is this “national approach” that relates to mortality rate.

All three investigators noted the quite different approaches between nations, as set out in their national guidelines. Some followed a “stay home” approach, whereas others defaulted to clinical assessment of patients either with COVID-19 or with any risk factor associated with it. For example, Singapore guidelines default to clinical assessment (26), whereas a country with a similar prevalence burden, the UK, has much higher thresholds for referral onward for assessment (27) (Table 2).

**Table 2:** Comparison of the criteria for assessment in suspected or confirmed COVID-19 between Singapore and the UK.

	**Singapore**	**UK**
**Criteria for clinical assessment**		
SpO_2_ (%)	<95	<92
Age (yrs)	>65	Irrelevant
Comorbidity	Any	Severe
Duration of illness (days)	>3	Irrelevant
**Epidemiology**		
Cases/million inhabitants	6,063	4,076
Physicians/10,000 head of capita	24	28
CFR (%)	0.08	13.4

*Information is based on the clinical guidelines from each nation and WHO (see Supplementary Material). SpO_2_–oxygen saturations. CFR, case fatality rate*.

In this situation, where the national guideline target SpO_2_ is part of an overall strategy of avoiding admissions, then whilst it does remain possible that conservative oxygen approaches do contribute to higher mortality, it may be the contribution of other policies to avoid admissions that leads to an increased CFR. In the UK vs. Singapore example, a target SpO_2_ of <92% is likely to be harmful, but equally, failing to account for age of the patient or duration of fever may also be harmful. As such, the relationship identified here between CFR and target SpO_2_ may be more a relationship between CFR and national strategy; target SpO_2_ may be more of an indicator of national policy.

### Limitations and Future Studies

This study highlights the variation in national guidelines for when to commence supplemental oxygen in patients with COVID-19. In of itself, this raises important questions as to the optimal response to COVID-19. Attempting to delineate the interventions and strategies that are potentially beneficial between nations is difficult without using a mortality estimation, which carries inherent confounders. CFR depends on many factors, not least of which is the accurate reporting of COVID-19-related deaths. Whilst we found no correlation between CFR and rates of testing or crude case burden, we could not account for disparities in reporting of deaths, nor did we analyse for differences in the age of the population, nor the socioeconomic status of the infected population.

We undertook an analysis of the national guidelines using three independent investigators. The consensus amongst the investigators supports the accuracy of the target SpO_2_ extracted. The possibility remains that localities within a country, or individual doctors and nurses, chose not to follow their national guidelines. Even if such local differences were significant, the national guidelines *permit* not implementing oxygen therapy until the target oxygen level is reached; therefore, triage systems, nurses, and physicians can avoid admissions and thus limit access to supplemental oxygen. Despite the prospect of local variations in following the guidelines, the presence of the guidelines will shape and likely reflect practise nationally.

Utilising patient-specific data from cohorts from the nations analysed here would provide greater insight into whether target oxygen saturation guidelines are both being followed and having a direct impact on mortality. A further follow-up study utilising patient-specific data could help determine whether the relationship identified here is in fact causal. It would also be useful to undertake further analysis into the cost-effectiveness of increasing access to supplemental oxygen vs. expanding intensive care facilities—could more lives be saved by treating more cases earlier and with optimum oxygen targets?

## Conclusion

There is clear disparity between national guidelines for target oxygen saturations (SpO_2_) in COVID-19 across the countries analysed here. Those nations that implemented a lower target oxygen level for when to commence oxygen in patients with COVID-19 had a significantly higher CFR. Whilst there are multiple confounders to the CFR, the overall relationship between increasing CFR with a decreasing target SpO_2_ warrants further investigation.

As it stands currently, our results support the position that managing COVID-19 pneumonia should not differ from the management of other pneumonias (24), insomuch as access to supplemental oxygen delivered optimally is necessary to prevent excessive mortality.

## Data Availability Statement

Publicly available datasets were analysed in this study. This data can be found at: https://www.who.int/docs/default-source/coronaviruse/situation-reports/20200518-covid-19-sitrep-119.pdf?sfvrsn=4bd9de25_4.

## Author Contributions

DG and FM initially conceived of the study. HD, AK, JN, and SB contributed further to the conception and/or design of the study and contributed to the manuscript. DG, HD, and AK conducted the analysis of the national guidelines. Statistical analysis was undertaken primarily by JN. FM and DG wrote the majority of the manuscript. All contributors reviewed the final manuscript before submission.

## Conflict of Interest

The authors declare that the research was conducted in the absence of any commercial or financial relationships that could be construed as a potential conflict of interest.
