# Supplementary material for: Oxygen and Mortality in COVID-19 Pneumonia: A Comparative Analysis of Supplemental Oxygen Policies and Health Outcomes Across 26 Countries
Source: Front Public Health. 2021 Jul 13;9:580585. doi: 10.3389/fpubh.2021.580585 (PMC8313806; doi:10.3389/fpubh.2021.580585)
Supplement: Supplementary file 1 [file Table_1.pdf]

Supplementary File

Oxygen and mortality in COVID-19 pneumonia: a comparative analysis of supplemental oxygen policies and health outcomes across 26 countries.

Goyal D, et al.,

	Target O2 Sats	Link to National Guidelines<br>All viewed between 18th May 2020 and 6th June 2020
**Pakistan**	94%	Government of Pakistan - guidelines for all patients with COVID-19<br><br>http://covid.gov.pk/new_guidelines/<br>05June2020_20200106_Clinical_Management_Guidelines_for_COVID-19_infection_v2.pdf
**Singapore**	94%	National Centre for Infectious Diseases, Singapore - guidelines for all patients with COVID-19<br><br>https://www.ncid.sg/Health-Professionals/Diseases-and-Conditions/Documents/<br>Treatment%20Guidelines%20for%20COVID-19%20%282%20Apr%202020%29%20-final.pdf
**Swiss**	94%	Swiss Society of Intensive Care, published in Swiss Medical Weekly DOI: https://doi.org/10.4414/<br>smw.2020.20227<br>Publication Date: 24.03.2020<br>Applies to all adult patients with COVID-19<br><br>https://smw.ch/article/doi/smw.2020.20227
**Ireland**	94%	Irish Thoracic Society.<br><br>https://irishthoracicsociety.com/wp-content/uploads/2020/03/COVID-Respiratory-Management-<br>Guideline09.04.20.pdf
**Qatar**	94%	Ministry of Public Health, Qatar<br><br>https://ddc.moph.go.th/viralpneumonia/eng/file/guidelines/g_CPG.pdf
**Saudi Arabia**	93%	Ministry of Health, Kingdom of Saudi Arabia<br><br>https://www.moh.gov.sa/Ministry/MediaCenter/Publications/Documents/Coronavirus-Disease-2019-<br>Guidelines-v1.2.pdf
**Chile**	93%	RECOMENDACIONES CLÍNICAS DE<br>KINESIOLOGÍA RESPIRATORIA EN ATENCIÓN DE<br>PACIENTES CON COVID-19<br>Sociedad Chilena de Kinesiología Respiratoria (SOCHIKIR)<br>Sociedad Argentina de Kinesiología Cardio Respiratoria (SAKICARE)<br>División de Kinesiología Intensiva, Sociedad Chilena de Medicina Intensiva (DIKISOCHIMI)<br><br>https://www.researchgate.net/publication/<br>340608875_Guia_de_recomendaciones_clinicas_de_kinesiologia_respiratoria_en_atencion_de_paci<br>entes_con_COVID-19
**India**	93%	INTERNATIONAL PULMONOLOGIST'S CONSENSUS ON COVID-19<br>https://www.unah.edu.hn/dmsdocument/9674-consenso-internacional-de-neumologos-sobre-<br>covid-19-version-ingles

	Target O2 Sats	Link to National Guidelines<br>All viewed between 18th May 2020 and 6th June 2020
**Portugal**	92%	SOCIEDADE PORTUGUESA DE PNEUMOLOGIA<br><br>RECOMENDAÇÕES DA SPP SOBRE TERAPIAS RESPIRATÓRIAS NÃO-INVASIVAS EM CONTEXTO DE DOENTE AGUDO/CRÓNICO AGUDIZADO NA COVID-19<br><br>https://www.sppneumologia.pt/uploads/subcanais_conteudos_ficheiros/terapias_spp.pdf
**Turkey**	92%	Turkey Ministry of Health<br>COVID-19 (SARS-CoV2 ENFEKSİYONU) REHBERİ, March 2020<br><br>https://covid19bilgi.saglik.gov.tr/depo/rehberler/COVID-19_Rehberi.pdf. Page 23
**Italy**	92%	Italian Thoracic Society (AIPO - ITS) and Italian Respiratory Society (SIP/IRS), March 2020<br>AIPO Managing the Respiratory care of patients with COVID-19.pdf<br><br>https://ers.app.box.com/s/j09ysr2kdhmkcu1ulm8y8dxnosm6yi0h
**UK**	91%	1. NHS England. Clinical guide for the optimal use of Oxygen therapy during the coronavirus pandemic. 9 April 2020 Version 1. https://www.england.nhs.uk/coronavirus/wp-content/uploads/sites/52/2020/04/C0256-specialty-guide-oxygen-therapy-and-coronavirus-9-april-2020.pdf<br>2. National Institute of Clinical Guidelines, UK - https://www.nice.org.uk/guidance/ng165
**Belgium**	91%	Institute of Tropical medicine Antwerp<br>INTERIM CLINICAL GUIDANCE FOR ADULTS WITH SUSPECTED OR CONFIRMED COVID-19 IN BELGIUM<br>Accessed 20/05/20<br>https://covid-19.sciensano.be/sites/default/files/Covid19/COVID-19_InterimGuidelines_Treatment_ENG.pdf
**France**	91%	Pneumology and Respiratory Intensive Care Department, Dijon University Hospital Center, Dijon, France<br>Procedure for the pulmonary management of non-ICU patients hospitalized in the context of the COVID-19 pandemic<br><br>http://splf.fr/wp-content/uploads/2020/04/RespiPreREA-SPLF-GAVO2avril2020-english-version-r.pdf
**Canada**	91%	Government of Canada<br>Clinical management of patients with moderate to severe COVID-19 - Interim guidance<br><br>https://www.canada.ca/en/public-health/services/diseases/2019-novel-coronavirus-infection/clinical-management-covid-19.html#5
**Peru**	94%	Society of Pneumologia, Peru<br>Lineamientos de manejo hospitalario del paciente con COVID-19, 28.MARZO.2020. http://www.spneumologia.org.pe https://drive.google.com/file/d/1VQk7Vo8mlJ4ilMekFzsW4rUOPIRy-8wX/view
**Brasil**	93%	GRUPO FORÇA COLABORATIVA COVID-19 BRASIL<br>Orientações sobre Diagnóstico, Tratamento e Isolamento de Pacientes com COVID-19.<br>Versão 01 Data:13/04/2020
**Germany**	90%	German Society of Medical Intensive Care and Emergency Medicine<br><br>German recommendations for critically ill patients with COVID-19<br><br>https://pneumologie.de/fileadmin/user_upload/COVID-19/German_recommendations_for_critically_ill_patients_with_COVID-19._MKIM_2019.pdf
**Iran**	92%	Health and Treatment Deputy of the Ministry of Health and Medical Education (2020). Guideline for the diagnosis and treatment of COVID-19 in outpatients and inpatients. http://dme.behdasht.gov.ir/uploads/Felo_Tashkish.pdf

	Target O2 Sats	Link to National Guidelines<br>All viewed between 18th May 2020 and 6th June 2020
**Bangladesh**	92%	Disease Control Division<br>Directorate General of Health Services Ministry of Health & Family Welfare Government of the People's Republic of Bangladesh<br><br>National Guidelines on Clinical Management of Coronavirus Disease 2019 (Covid-19) Version 4.0 30 March 2020<br><br>http://www.mohfw.gov.bd/index.php?option=com_docman&task=doc_download&gid=22424&lang=en.
**China**	91%	Jin YH, Cai L, Cheng ZS, et al. A rapid advice guideline for the diagnosis and treatment of 2019 novel coronavirus (2019-nCoV) infected pneumonia (standard version). Mil Med Res. 2020;7(1):4. Published 2020 Feb 6. doi:10.1186/s40779-020-0233-6
**USA**	91%	Poston JT, Patel BK, Davis AM. Management of Critically Ill Adults With COVID-19. JAMA. Published online March 26, 2020. doi:10.1001/jama.2020.4914
**Spain**	90%	SOCIEDAD ESPAÑOLA DE NEUMOLOGÍA Y CIRUGÍA TORÁCICA<br><br>FISIOTERAPIA RESPIRATORIA EN EL MANEJO DEL PACIENTE CON COVID-19: RECOMENDACIONES GENERALES<br><br>Spain - http://svmefr.com/wp-content/uploads/2020/03/COVID19-SEPAR-26_03_20.pdfhttp://svmefr.com/wp-content/uploads/2020/03/COVID19-SEPAR-26_03_20.pdf
**Mexico**	90%	Government of Mexico<br><br>https://coronavirus.gob.mx/wp-content/uploads/2020/04/Flujograma_Atencion_Primer_Nivel_13042020.pdf
**Sweden**	90%	Drug Therapeutic Committee and the Health and Medical Care Administration of the Stockholm County Council, Sweden<br><br>https://janusinfo.se/behandling/akutinternmedicin/infektionssjukdomar/infektionssjukdomar/covid19infektion.5.5d5ae8ba1719cea8d541290e.html#h-Skyddsutrustninginomsjukvarden
**Russia**	93%	Ministry of Health<br><br>ПРОФИЛАКТИКА,<br>ДИАГНОСТИКА И ЛЕЧЕНИЕ НОВОЙ КОРОНАВИРУСНОЙ ИНФЕКЦИИ<br><br>https://static-1.rosminzdrav.ru/system/attachments/attaches/000/050/116/original/28042020_MR_COVID-19_v6.pdf

Supplementary Table 1. Target oxygen saturations with links to national guidelines.
